# Influence of genetic background on the clinical picture of bipolar affective disorder in a population of children and adolescents

**DOI:** 10.1192/j.eurpsy.2024.959

**Published:** 2024-08-27

**Authors:** K. Kamińska, M. Bień, K. Dąbrowska, M. Janas-Kozik, L. Cichoń, K. Wilczyński

**Affiliations:** Medical University of Silesia, Katowice, Poland

## Abstract

**Introduction:**

Bipolar disorder in children is characterized by a different course than in adults, which is a diagnostic difficulty. DAT-1 is a dopamine transporter gene that regulates dopaminergic neurotransmission through the mechanism of active reuptake of this neurotransmitter from the synapse. Polymorphisms within the described gene can result in changes in dopamine levels, which may have implications for the development of bipolar disorder.

**Objectives:**

The aim of the project was to analyze the relationship between single nucleotide polymorphisms (SNPs) within the dopamine transporter gene DAT-1 and the risk of development of bipolar disorder in a population of children and adolescents.

**Methods:**

21 healthy controls (12 females, 9 males) have been recruited into the study and 13 patients (9 girls, 4 boys) with bipolar disorder diagnosis from Department of Psychiatry and outpatient clinic, were recruited for the study group. Questionnaires such as the KSADS-PL were carried out and blood was taken for laboratory tests of four SNPs within the DAT-1 transporter. PQStat, Microsoft Excel 2013 and StatSoft STATISTICA were used to perform the statistical analysis.

**Results:**

SNPs within the dopamine transporter gene and environmental risk factors influenced the risk of developing bipolar disorder in the population of children and adolescents.

**Conclusions:**

The ambiguity in results emphasizes the necessity for further investigations into correlation between genetic factors in bipolar disorder etiology. Future research should involve more participants. The results of this project are likely to make a significant and valuable contribution to the current knowledge of bipolar disorder and to the development of innovative diagnostic methods, making a significant contribution to the advancement of science.

**Disclosure of Interest:**

None Declared

